# New Laboratory Furnace

**Published:** 1883-08

**Authors:** 


### NEW LABORATORY FURNACE.

One of the most convenient appliances which we have ever used is a small laboratory gas-furnace, made by Dr. C. B. Parker, of Brooklyn. A cylinder of heavy sheet iron, about six inches high and seven in diameter, a little larger at the top than at the bottom, is so lined with fire-clay, or with asbestos and plaster of paris, as to present a flue, constricted just above the middle, and with a bell shaped top. Across the summit of the narrowest part, with their ends embedded in the lining, are arranged parallel iron bars, upon which may be placed a properly invested gold plate which is to be heated up, or which will hold a crucible containing metal for melting. Above these, in the wider portion, are placed movable bars intended to support casts while drying, or to hold ladles for melting zinc or tin for swages, or upon which may be placed a basin for heating water. The whole is supported by a tripod, and is intended to be placed over a Fletcher-blast gas-burner (No. 7, Buffalo Dental Manufacturing Co.'s catalogue of Fletcher's Labaratory apparatus).

The laboratory uses to which this simple furnace is adapted are almost endless. The heat is readily controlled by limiting the flow of gas, while by the use of the foot bellows even refractory metals readily yield. In the soldering of gold plates it is especially convenient, as the whole plate may be kept at a red heat, the soldering being done without removing the piece from the furnace, and thus all danger of warping the plate or of cracking the teeth, is obviated, to say nothing of the time saved.

The manufacture of it is in no way restricted, Dr. Parker desiring that any benefits to be derived from its use shall be free to all.

